# Undernutrition among rural school-age children: a major public health challenge for an aspirational district in Karnataka, India

**DOI:** 10.3389/fnut.2023.1209949

**Published:** 2023-07-12

**Authors:** Phaniraj Vastrad, Sushrit Neelopant, U. Venkateswara Prasad, Rahul Kirte, N. Chandan, Manish J. Barvaliya, Shivappa Hatnoor, S. B. Shashidhar, Subarna Roy

**Affiliations:** ^1^Model Rural Health Research Unit, Department of Health Research (Government of India), Sirwar, Raichur, Karnataka, India; ^2^Raichur Institute of Medical Sciences, Raichur, Karnataka, India; ^3^National Institute of Traditional Medicine, Indian Council of Medical Research (ICMR), Belagavi, Karnataka, India

**Keywords:** anthropometry, undernutrition, school-age children, stunting, thinness, BMI

## Abstract

**Background:**

For school-age children, a healthy transition from childhood to adolescence and adulthood depends on proper nutrition. Globally, most nutritional surveys focus on preschool and adolescents, neglecting school-age children. Recent studies have shown the prevalence of thinness among adolescents to be 26.5% in Karnataka. Similarly, among children aged < 5 years in the Raichur district, the prevalence of stunting, wasting, and being underweight was 39.8, 23.2, and 40.7%, respectively. The present study aimed to bridge the data gap between < 5 years of children and adolescents through a nutritional survey of school-going children in Raichur, one of the aspirational districts of India.

**Materials and methods:**

A cross-sectional survey was conducted from January to March 2020 among rural school-age children (*n* = 2700) in 30 villages of the Raichur district of Karnataka, India. The school children were selected through a multi-stage cluster sampling technique. The WHO Anthro-plus software was used for calculating the age and sex-specific Z-scores for weight-for-age (WAZ), height-for-age (HAZ), and BMI-for-age (BAZ).

**Results:**

Of the 2,700 school-age children surveyed, the mean weight and height were 22.2 kg (+5.8) and 124.9 cm (+11.6), respectively. The prevalence of children having weight-for-age Z-scores < −2 SD (Underweight) was 45.3% (95% CI: 42.7%−47.8%). The magnitude of stunting and severe stunting was 19.5% (95% CI: 18.0%−21.0%) and 7.6% (95% CI: 6.6%−8.6%), respectively. The proportion of children with BMI for age Z-scores < −2SD (thinness) was 43% (95% CI: 41.1%−44.9%), with sub-district Sindhanur having a dual burden of malnutrition.

**Conclusion:**

Despite many flagship programs, the prevalence of undernutrition in school-age children remains a considerable public health problem in the aspirational district of Raichur, India. Furthermore, exploratory studies are recommended to identify the factors associated with undernutrition among school-age children and strategize evidence-based intervention.

## Introduction/background

Undernutrition is still a significant health challenge globally, especially in low- and middle-income countries (1). Undernutrition comprises four forms: stunting (low height-for-age), wasting (low weight for height), underweight (low weight-for-age), and micronutrient deficiencies. Globally in 2020, 149 million children were stunted, 45 million were estimated to be wasted, and approximately 45% of deaths among children below the age of 5 years were associated with undernutrition (2). A recently conducted National Family Health Survey-5 in India during 2019–2020 estimated that 35%, 19.3%, and 32.1% of children younger than 5 years were stunted, wasted, and underweight, respectively (3). Despite several large-scale flagship programs such as the Mid-Day Meal Scheme, Integrated Child Development Services (ICDS), and Prime Minister's Overarching Scheme for Holistic Nutrition (POSHAN), the persistence of undernutrition in India is impeding the efforts to achieve the targets set by the National Nutrition Mission in which the aim is to reduce the stunting in children (0–6 years of age) from 38.4% to 25% by 2022 (Mission 25 by 2022) in India (4, 5).

Undernourished children have a high risk of infection due to immune dysfunction (6, 7), growth flattering (8), neurocognitive impairment, decreased physical capacity, poor academic performance, increased absenteeism, and early school dropout (7, 9–11). While monitoring the nutritional indicators among children younger than 5 years has always been key in the Millennium Development Goals, the Sustainable Development Goals, and the NFHS-5 survey in India (3, 12, 13), it has inadvertently led to overlooking nutritional status among school-age children. Older children and adolescents are mainly at risk of undernutrition because they experience a growth spurt, requiring all essential macronutrients and micronutrients to supplement the body's increased demand for attaining puberty and development. Many studies have also documented that undernutrition is associated with delayed sexual maturation, which is required to achieve an adult's developmental potential (14, 15).

Globally, most nutritional surveys primarily focus on children of < 5 years and adolescents (11–15), neglecting school-going children, which have been highlighted in a few studies conducted in India and African countries (7, 10, 16). A nutritional survey of school-going children is essential in understanding their transition from childhood to adolescence and adulthood (14–17). The preschool years are the first crucial time for intervention when a child's overall growth is compromised. However, some studies have shown evidence of catch-up growth beyond the preschool age. With the right nutritional interventions, school-going children who are undernourished have a significantly high chance of catching up during the pubertal period (the second crucial time) (15, 18, 19). The World Health Organization (WHO) added growth standards for children aged 5 to 19 years in 2007 by using the prevalence of stunting, underweight, and thinness as undernutrition indicators rather than wasting in recognition of the importance of the age group and to close the gap (20). Despite that, nutritional data on school-aged children are globally scarce, including in India (21, 22).

India is a large nation with a diverse geography, climatic conditions, socioeconomic distribution, religion, culture, and food habits. Thus, the prevalence of undernutrition differs substantially between the states and districts. NITI Aayog of the Government of India introduced the Aspirational Districts Programme in 2018 to catalyze development in the 112 most backward districts of the country (23, 24). In Karnataka, Raichur and Yadgir are the only two districts in the Aspirational Districts Programme. The program aims to rapidly and sustainably transform the backward districts to catch up with the rest of the country in terms of key development indicators. The Aspirational Districts Programme is a holistic effort to address all aspects of development, including health and nutrition. The introduction of the Aspirational Districts Programme recognizes that despite decades of action, there are still significant disparities in the development across India. The program will go a long way in ensuring that the fruits of development reach all sections of society, especially the most vulnerable and marginalized (24). Raichur district has a low literacy rate of 59.56%, indicating limited educational attainment (25). The NITI Aayog report highlights that 32.19% of the population in Raichur experiences multidimensional poverty, emphasizing the prevalence of socioeconomic challenges in the region (26).

The prevalence of stunting, wasting, and being underweight among children aged < 5 years was 39.8, 23.2, and 40.7%, respectively, in the Raichur district (27). The prevalence of thinness among adolescents was 26.5% in the state of Karnataka (28). There is a need to bridge the data gap regarding the growth and nutritional status between children of age < 5 years and adolescents. Hence, the present study was designed to evaluate the nutritional status among school-age children in one of the aspirational districts of India.

## Materials and methods

### Study design

The present cross-sectional study was conducted on rural school-going children (6–12 years), which was chosen using a multi-stage cluster sampling method. It was a part of the Iodine Deficiency Disorder survey conducted in the rural areas of the Raichur district from January to March 2020. The survey was solicited by the Deputy Director of Nutrition, Directorate of Health and Family Welfare Services, Government of Karnataka.

### Study setting and participants

Five clusters/sub-districts covering 30 villages in the Raichur district, Karnataka, were included in the study. The number of villages covered in each sub-district/taluka was; Devadurga 5, Lingasugur 7, Manvi 7, Raichur 6, and Sindhanur 5 villages. The children of age group 6–12 years studying in the government primary schools of the selected villages were included in the study.

### Sample size and sampling techniques

**Figure 1:**
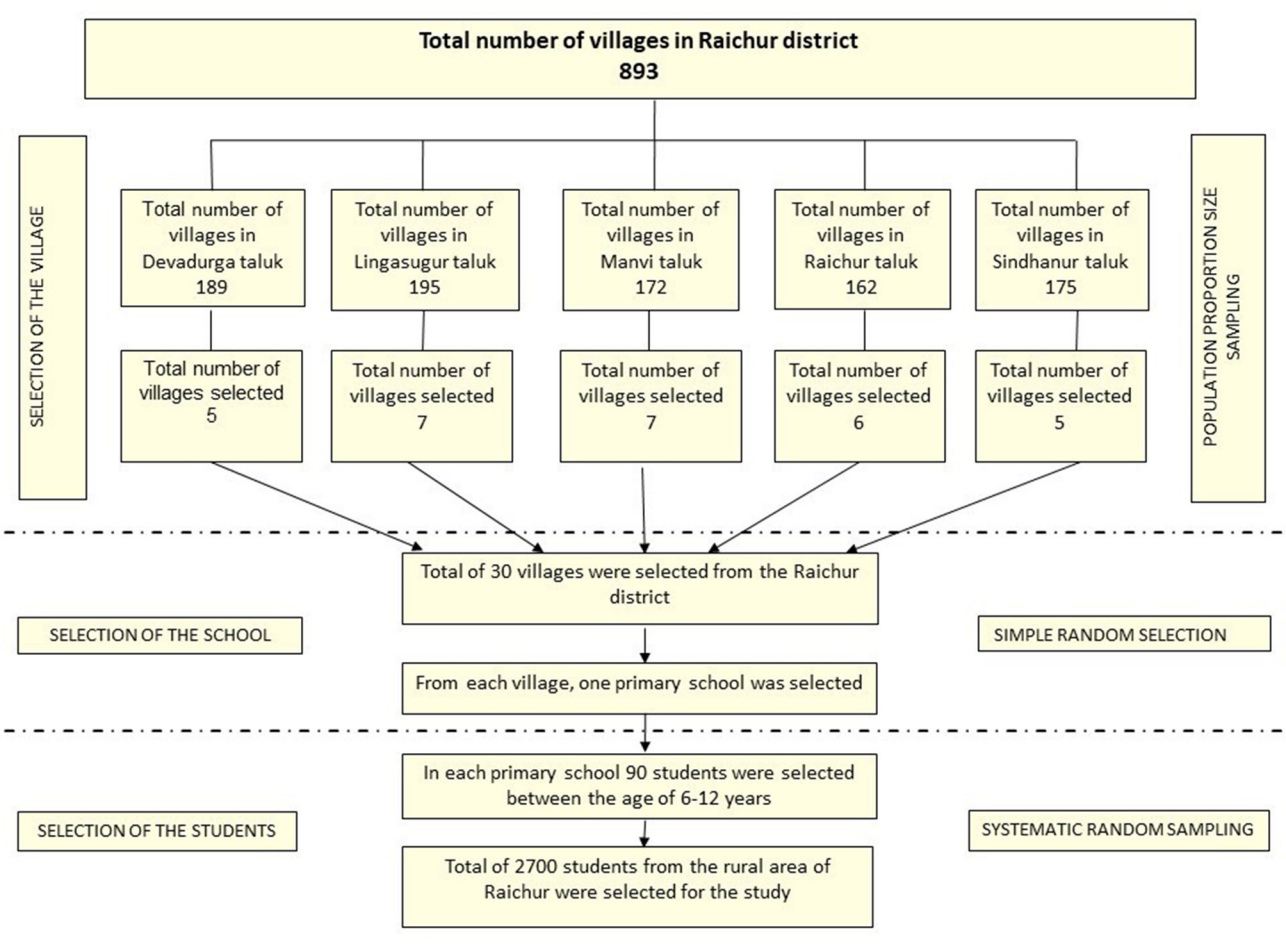
Flowchart summarizing the sampling process adopted during the study.

This study was a part of the Iodine Deficiency Disorders (IDD) survey conducted under the National Iodine Deficiency Disorders Control Programme (NIDDCP). Thus, followed the sample size and sampling method as per Revised NIDDCP guidelines (29). No formal sample size estimation was carried out for the study. The samples were selected using a multi-stage cluster sampling method with probability proportional to size. The survey involved a sample size of 2,700 students, comprising 90 children from each village. Figure 1 describes the flow of sampling conducted during the study.

### Selection of the villages using the PPS method

A total of 30 villages from the five sub-districts/talukas of the Raichur district were selected using the PPS systematic sampling method. Initially, the complete list of villages and the population were obtained from the Zilla Panchayat Office, Raichur. Later, the villages were selected after calculating sampling intervals, and the first village was chosen randomly. The following steps were involved during the selection process of the villages.

1. Number of villages = 30
2. Calculation of sampling interval
$$\begin{array}{l} =\frac{\text{Total population of the district}}{30}=\text{“k”} \end{array}$$
3. Selection of random start between 1 to k, say “n”
4. Selected random numbers were n, n+k, n+2k,............... n+29k.

Finally, five villages from Devadurga, seven from Lingasugur, seven from Manvi, six from Raichur, and five from Sindhanur were included.

### Selection of the school in the village by simple random sampling

Many of the chosen villages had one primary school; in the villages with more than one primary school, the school was selected randomly by the balloting method.

### Selection of the students from schools by systematic random sampling

On the day of the visit, the list of students (6–12 years) present was obtained from the attendance register of the school. The students in each school were stratified by their grades and gender. Both male and female children were included proportionately in a 1:1 ratio. Later, 90 students allocated to the school were distributed to six participating grades, proportionately based on the student population in the class. Finally, students were selected in each stratum using a systematic random sampling technique.

### Ethical consideration

The study documents were reviewed and approved by the Institutional Ethics Committee of Raichur Institute of Medical Sciences (RIMS), with approval number RIMS/IEC/Approval/02/22–23. During the survey, written consent from the parents/guardians of the students was obtained. Verbal assent was taken from the children between the age of 6 and 12 years. Before starting the survey, permission was sought from the concerned government officials.

### Anthropometric data collection

The data were collected by the qualified and trained doctors of RIMS and a medical scientist from the Model Rural Health Research Unit (Indian Council of Medical Research—National Institute of Traditional Medicine) using a pretested, structured questionnaire and a checklist to record anthropometric measurements. Before the survey, the data collecting team was trained at RIMS, Raichur, regarding data collection, anthropometric measurements, and clinical assessment. Weights in kilograms were recorded to the nearest 100 g (0.1 kg) using SECA^®^813 digital flat scale. Similarly, height was measured in centimeters to the nearest 1 mm (0.1 cm) using SECA portable stadiometer model 213. To minimize error, the height and weight of each child were measured thrice, and the average was considered. Using a standard 10 kg weight, the weighing scale was checked for accuracy and consistency each day before commencing the survey. While measuring the height, the children were asked to stand on the stadiometer in such a way that their heels, buttocks, and heads touched the vertical stand of the stadiometer. The heads of the students were positioned at the Frankfurt Horizontal Line/Eye Ear line to ensure uniformity. Later, the anonymized data were entered into Microsoft Excel for further analysis.

### Data analysis

The data were imported into SPSS software (IBM SPSS Statistics for Windows, version 25) and were analyzed for descriptive statistics. Two-sample Z-test of proportions was performed to determine any significant difference in age and gender characteristics. *Z*-scores were computed using the WHO Anthro-plus software for weight-for-age (WAZ), height-for-age (HAZ), and BMI for age (BAZ). Underweight, stunting, and thinness were assessed using WAZ, HAZ, and BAZ, respectively, based on the WHO growth reference 2007 criteria for measuring malnutrition in school-age children (20). Z-score values below−2SD were considered underweight, thin, and stunted. Z-scores between−2SD and−3SD were considered moderate, whereas < −3SD was considered severe. BAZ greater than +1SD and less than +2SD was referred to as overweight, +2SD to +3SD as obese, and > +3SD was defined as severely obese. The WAZ was calculated up to the age of 9 years (119 months), as it is insufficient for growth monitoring beyond 10 years due to its inability to differentiate between relative height and body mass. The distribution plots of Z-scores based on age, sex, and cluster (taluka) compared with WHO child growth standards were also generated using WHO Anthro-plus software.

## Results

**Figure 2:**
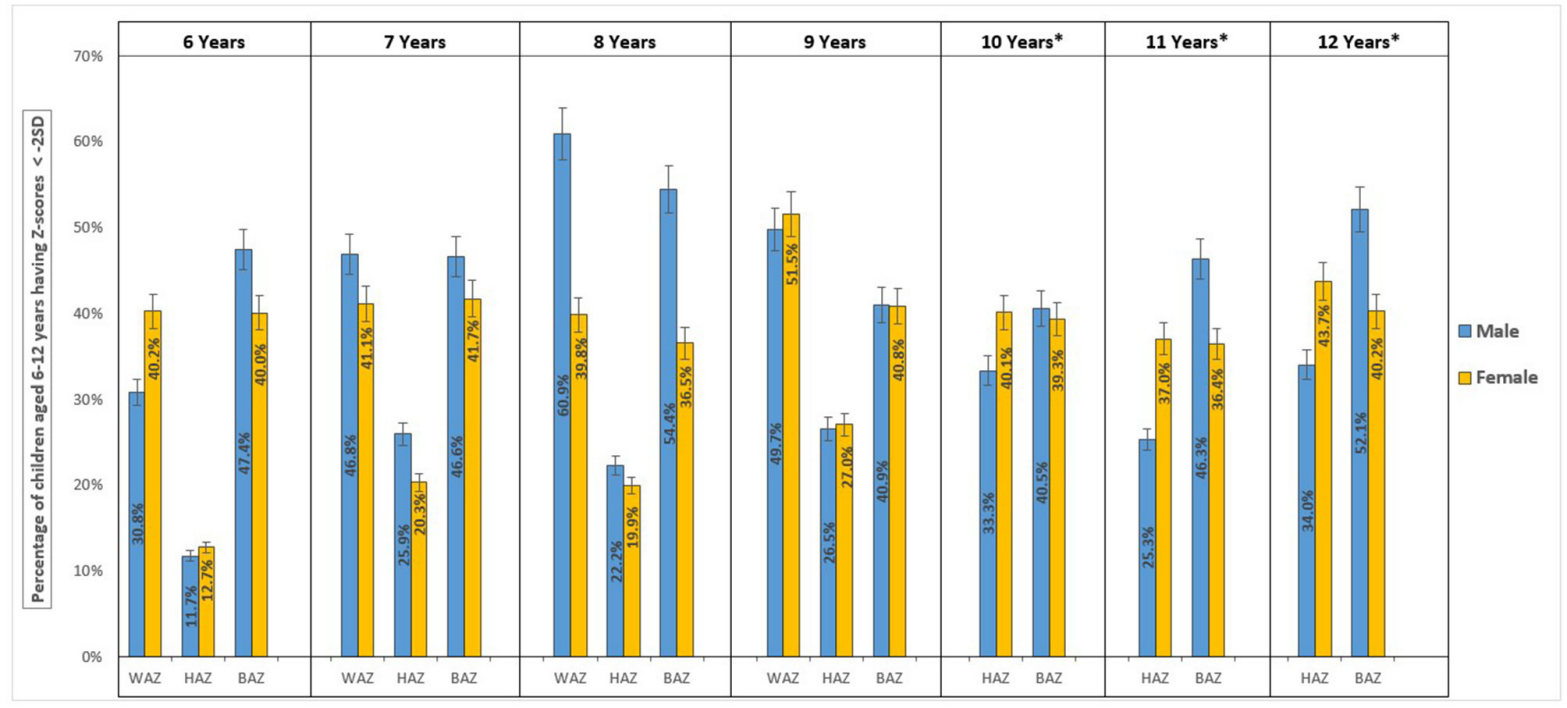
Prevalence of undernutrition, stunting, and thinness among school age children (6–12 years) categorized by age and gender.

**Figure 3:**
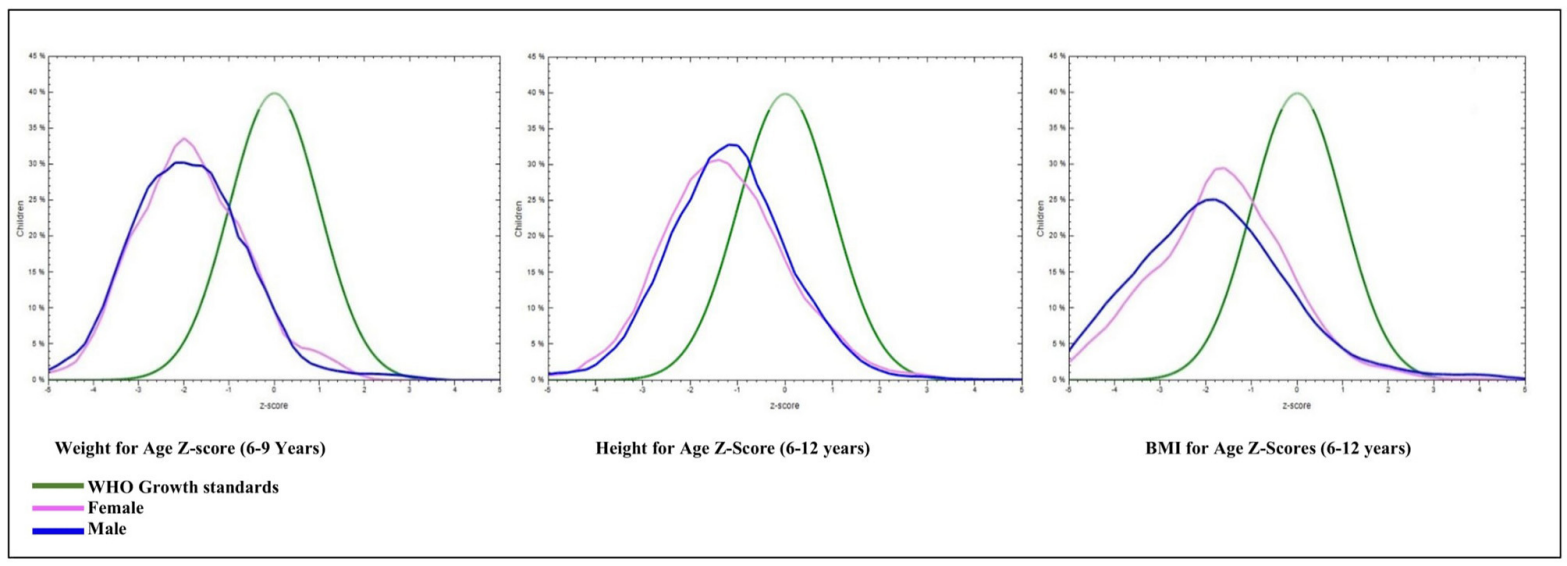
Kernel density plots displaying *Z*-score distribution of Weight-for-Age, Height-for-Age, and BMI- for-Age by gender compared with WHO reference 2007 (61 months to 19 years) normal distribution curve.

The study included 2,700 rural school students from 30 different schools in the Raichur district of Karnataka. After data cleaning, measurements for weight and height were available for 2,602 (96.4%) and 2,688 (99.5%) students, respectively. The age of children ranged from 72 to 155 months. There was an even distribution of male (1,349; 49.9%) and female (1,351; 50.1%) school children for the study. The mean weight and height of the study sample were 22.2 kg (+5.8) and 124.9 cm (+11.6), respectively (Table 1). Figure 2 illustrates the prevalence of underweight, stunting, and thinness categorized by age and gender. The kernel density plots for WAZ, HAZ, and BAZ portrayed skewness toward the left side when compared with the WHO growth standard graph (Figure 3).

**Table 1:** Characteristics of the school children surveyed in the rural areas of the Raichur district.

**Characteristics**	**Number (%)**
**Age (*****n** =* **2,700)**	
6 years	373 (13.8%)
7 years	381 (14.1%)
8 years	398 (14.7%)
9 years	392 (14.5%)
10 years	386 (14.3%)
11 years	379 (14.0%)
12 years	391 (14.5%)
**Gender (*****n** =* **2,700)**	
Male	1,349 (49.9)
Female	1,351 (50.1)
Height (cm) (Mean +SD)	124.9 + 11.6
Weight (kg) (Mean +SD)	22.2 + 5.8
**Cluster/Taluka (*****n** =* **2,700)**	
Manvi	630 (23.3%)
Devadurga	450 (16.7%)
Lingasur	630 (23.3%)
Raichur	540 (20.0%)
Sindhanur	450 (16.7%)
**Weight for Age (Underweight) (*****n** =* **1,540)**	
Normal	843 (54.7%)
Moderate underweight	423 (27.5%)
Severe underweight	274 (17.8%)
**Height for Age (Stunting) (*****n** =* **2,688)**	
Normal	1,958 (72.8%)
Moderate stunting	525 (19.5%)
Severe stunting	205 (7.6%)
**BMI for Age (Thinness) (*****n** =* **2,602)**	
Severe Thinness	556 (21.4%)
Thinness	563 (21.6%)
Normal BMI	1,371 (52.7%)
Overweight	65 (2.5%)
Obese	26 (1%)
Severely Obese	21 (0.8%)

### Weight-for-age

Of the 1,540 total samples, 698 (45.3% (95% CI: 42.7%−47.8%) children had a WAZ score below−2 SD (underweight). The prevalence of severe underweight among the children was 17.8% (95% CI: 15.8%−19.7%). Male children were more likely to be underweight at 47.4% (95% CI: 43.7%, 51%) compared with female children at 43.2% (95% CI: 39.7%−46.8%); however, the difference was not statistically significant (*p*-value = 0.098). In male children, the prevalence of underweight was high in the age group of 96–107 months 60.9% (95% CI: 53.8–68%); whereas in female children, it was high in 108–119 months, 51.5% (95% CI: 44.3–58.7%); of age (Figure 2; Table 2).

**Table 2:** Distribution of weight-for-age among the school children of age 6–9 years in the rural areas of Raichur district, Karnataka, India.

**Age groups**			**Weight-for-age** ^**^ **(%)**					
**Years**	**Months**	**N**	**% < −3SD**	**(95% CI)**	**% < −2SD^1^**	**(95% CI)**	**Mean**	**SD**
**Male**								
6	(72–83)	182	14.3	(8.9%, 19.6%)	30.8	(23.8%, 37.7%)	−1.47	1.41
7	(84–95)	188	17.6	(11.8%, 23.3%)	46.8	(39.4%, 54.2%)	−1.96	1.25
8	(96–107)	197	30.5	(23.8%, 37.1%)	60.9	(53.8%, 68%)	−2.24	1.25
9	(108–119)	191	15.2	(9.8%, 20.5%)	49.7	(42.4%, 57.1%)	−1.92	1.21
Total (6–9)	(72–119)	758	19.5	(16.6%, 22.4%)	47.4	(43.7%, 51%)	–1.91	1.31
**Female**								
6	(72–83)	189	6.9	(3%, 10.8%)	40.2	(33%, 47.5%)	−1.49	1.15
7	(84–95)	192	10.9	(6.3%, 15.6%)	41.1	(33.9%, 48.4%)	−1.77	1.19
8	(96–107)	201	19.4	(13.7%, 25.1%)	39.8	(32.8%, 46.8%)	−1.74	1.2
9	(108–119)	200	26.5	(20.1%, 32.9%)	51.5	(44.3%, 58.7%)	−2	1.35
Total (6–9)	(72–119)	782	16.1	(13.5%, 18.8%)	43.2	(39.7%, 46.8%)	–1.75	1.24
**Both Sexes Combined**								
6	(72–83)	371	10.5	(7.3%, 13.8%)	35.6	(30.6%, 40.6%)	−1.48	1.28
7	(84–95)	380	14.2	(10.6%, 17.9%)	43.9	(38.8%, 49.1%)	−1.86	1.22
8	(96–107)	398	24.9	(20.5%, 29.2%)	50.3	(45.2%, 55.3%)	−1.99	1.25
9	(108–119)	391	21	(16.8%, 25.1%)	50.6	(45.6%, 55.7%)	−1.96	1.28
Total (6–9)	(72–119)	1540	17.8	(15.8%, 19.7%)	45.3	(42.7%, 47.8%)	–1.83	1.27

1 Includes children who are below−3 SD from the WHO Child Growth Standards.

** Weight-for-age reference data are not available beyond 10 years because this indicator does not distinguish between height and body mass in an age period where many children are experiencing the pubertal growth spurt and may appear as having excess weight (by weight-for-age) when in fact they are just tall.

### Height-for-age

Among 2,688 school children, stunting (HAZ score below−2 SD) was found in 27.2% (95% CI: 25.5%−28.9%), and the proportion of severe stunting was 7.6% (95% CI: 6.6%−8.6%). Female children were marginally more stunted as compared with male school children (28.6% (95% CI: 26.1%−31%) vs. (25.7% (95% CI: 23.4%−28.1%) but not statistically significant (*p*-value = 0.0841). Within the age group, stunting was high in the age group of 144–155 months in both male (34.0%) and female (43.7%) school children, and the difference between the genders was significant with a *p*-value of < 0.001 (Table 3). The graph generated from the WHO AnthroPlus has also shown the distribution of the HAZ scores among the school children shifted toward the left compared with the WHO growth standard graph (Figure 3).

**Table 3:** Distribution of height-for-age among the school children of age 6–12 years in the rural areas of Raichur district, Karnataka, India.

**Age groups**			**Height-for-age (%)**					
**Years**	**Months**	**N**	**% < −3SD**	**(95% CI)**	**% < −2SD^1^**	**(95% CI)**	**Mean**	**SD**
**Male**								
6	(72–83)	180	2.2	(0%, 4.7%)	11.7	(6.7%, 16.6%)	−0.49	1.41
7	(84–95)	189	7.4	(3.4%, 11.4%)	25.9	(19.4%, 32.4%)	−0.99	1.53
8	(96–107)	194	5.7	(2.2%, 9.2%)	22.2	(16.1%, 28.3%)	−1.1	1.34
9	(108–119)	189	6.3	(2.6%, 10.1%)	26.5	(19.9%, 33%)	−1.25	1.15
10	(120–131)	198	7.1	(3.2%, 10.9%)	33.3	(26.5%, 40.2%)	−1.32	1.25
11	(132–143)	190	3.7	(0.7%, 6.6%)	25.3	(18.8%, 31.7%)	−1.41	1.15
12	(144–155)	200	13.5	(8.5%, 18.5%)	34	(27.2%, 40.8%)	−1.79	1.09
Total (6–12)	(72–155)	1340	6.6	(5.3%, 8%)	25.7	(23.4%, 28.1%)	–1.21	1.33
**Female**								
6	(72–83)	189	3.7	(0.7%, 6.7%)	12.7	(7.7%, 17.7%)	−0.66	1.33
7	(84–95)	192	4.7	(1.4%, 7.9%)	20.3	(14.4%, 26.3%)	−0.85	1.37
8	(96–107)	201	6.5	(2.8%, 10.1%)	19.9	(14.1%, 25.7%)	−0.99	1.45
9	(108–119)	200	4	(1%, 7%)	27	(20.6%, 33.4%)	−1.19	1.14
10	(120–131)	187	12.3	(7.3%, 17.3%)	40.1	(32.8%, 47.4%)	−1.7	1.25
11	(132–143)	189	12.2	(7.2%, 17.1%)	37	(29.9%, 44.2%)	−1.6	1.28
12	(144–155)	190	17.4	(11.7%, 23%)	43.7	(36.4%, 51%)	−1.92	1.17
Total (6–12)	(72–155)	1348	8.6	(7.1%, 10.1%)	28.6	(26.1%, 31%)	–1.27	1.36
**Both Sexes Combined**								
6	(72–83)	369	3	(1.1%, 4.9%)	12.2	(8.7%, 15.7%)	−0.58	1.37
7	(84–95)	381	6	(3.5%, 8.6%)	23.1	(18.7%, 27.5%)	−0.92	1.45
8	(96–107)	395	6.1	(3.6%, 8.6%)	21	(16.9%, 25.2%)	−1.04	1.4
9	(108–119)	389	5.1	(2.8%, 7.5%)	26.7	(22.2%, 31.3%)	−1.22	1.14
10	(120–131)	385	9.6	(6.5%, 12.7%)	36.6	(31.7%, 41.6%)	−1.51	1.26
11	(132–143)	379	7.9	(5.1%, 10.8%)	31.1	(26.3%, 35.9%)	−1.51	1.22
12	(144–155)	390	15.4	(11.7%, 19.1%)	38.7	(33.8%, 43.7%)	−1.85	1.13
Total (6–12)	(72–155)	2688	7.6	(6.6%, 8.6%)	27.2	(25.5%, 28.9%)	–1.24	1.34

1 Includes children who are below−3 SD from the WHO Child Growth Standards.

### BMI-for-age

The prevalence of severe thinness, thinness, overweight, obesity, and severe obesity in school-age children was shown by the distribution of BAZ scores in the study samples to be 21.4% (95% CI: 19.8%−23%), 21.6% (95% CI: 20.1%−23.2%), 2.5% (95% CI: 1.9%−3.1%), 1% (95% CI: 0.6%−1.4%), and 0.8% (95% CI: 0.5%−1.2%), respectively. In male and female children combined, the prevalence of thinness [(46.2%) (41%−51.3%)] was high in the age group of 144–155 months. The study's findings depicted a statistically significant (*p*-value = 0.0001) higher prevalence of thinness in male children [(46.8%) (95% CI: 44.1%−49.6%)] than in female children [(39.3%) (95% CI: 36.6%−41.9%)]. In male children of the age group of 96–107 months, the prevalence of thinness was much higher [(54.4% (95% CI: 46.9%−61.9%)] compared with the other age groups. Similarly, in female children, the prevalence of thinness [(41.7%) (95% CI: 34.4%−49%]) was high in the age group of 84–95 months (Figure 2; Table 4).

**Table 4:** Distribution of BMI-for-age among the school children of age 6–12 years in the rural areas of Raichur district, Karnataka, India.

**Age groups**			**BMI-for-age (%)**											
**Years**	**Months**	**N**	**%** < −**3SD**	**(95% CI)**	**%** < −**2SD**^1^	**(95% CI)**	**%** > +**1SD**	**(95% CI)**	**%** > +**2SD**	**(95% CI)**	**%** > +**3SD**	**(95% CI)**	**Mean**	**SD**
**Male**														
6	(72–83)	171	25.1	(18.4%, 31.9%)	47.4	(39.6%, 55.1%)	7	(2.9%, 11.1%)	5.8	(2%, 9.7%)	3.5	(0.5%, 6.6%)	–1.72	1.85
7	(84–95)	176	25	(18.3%, 31.7%)	46.6	(38.9%, 54.2%)	3.4	(0.4%, 6.4%)	1.7	(0%, 3.9%)	1.1	(0%, 3%)	–1.9	1.66
8	(96–107)	182	30.8	(23.8%, 37.7%)	54.4	(46.9%, 61.9%)	3.8	(0.8%, 6.9%)	1.6	(0%, 3.8%)	0.5	(0%, 1.9%)	–2.12	1.64
9	(108–119)	181	19.3	(13.3%, 25.4%)	40.9	(33.4%, 48.3%)	6.6	(2.7%, 10.5%)	3.3	(0.4%, 6.2%)	2.8	(0.1%, 5.4%)	–1.56	1.75
10	(120–131)	195	21	(15%, 27%)	40.5	(33.4%, 47.7%)	7.2	(3.3%, 11.1%)	3.1	(0.4%, 5.8%)	1	(0%, 2.7%)	–1.57	1.73
11	(132–143)	188	21.3	(15.2%, 27.4%)	46.3	(38.9%, 53.7%)	4.8	(1.5%, 8.1%)	1.6	(0%, 3.7%)	1.1	(0%, 2.8%)	–1.8	1.55
12	(144–155)	190	26.8	(20.3%, 33.4%)	52.1	(44.7%, 59.5%)	3.7	(0.7%, 6.6%)	0.5	(0%, 1.8%)	0	(0%, 0.3%)	–1.97	1.5
Total (6–12)	(72–155)	1283	24.2	(21.8%, 26.5%)	46.8	(44.1%, 49.6%)	5.2	(4%, 6.5%)	2.5	(1.6%, 3.4%)	1.4	(0.7%, 2.1%)	−1.81	1.68
**Female**														
6	(72–83)	185	18.9	(13%, 24.8%)	40	(32.7%, 47.3%)	5.9	(2.3%, 9.6%)	3.2	(0.4%, 6.1%)	1.1	(0%, 2.8%)	–1.57	1.61
7	(84–95)	187	20.3	(14.3%, 26.4%)	41.7	(34.4%, 49%)	2.1	(0%, 4.5%)	0	(0%, 0.3%)	0	(0%, 0.3%)	–1.78	1.36
8	(96–107)	197	17.8	(12.2%, 23.4%)	36.5	(29.6%, 43.5%)	3	(0.4%, 5.7%)	1.5	(0%, 3.5%)	0	(0%, 0.3%)	–1.63	1.43
9	(108–119)	191	20.9	(14.9%, 27%)	40.8	(33.6%, 48.1%)	4.7	(1.4%, 8%)	1	(0%, 2.8%)	0	(0%, 0.3%)	–1.73	1.6
10	(120–131)	183	16.9	(11.2%, 22.6%)	39.3	(32%, 46.7%)	1.6	(0%, 3.8%)	0.5	(0%, 1.9%)	0	(0%, 0.3%)	–1.63	1.4
11	(132–143)	187	17.6	(11.9%, 23.4%)	36.4	(29.2%, 43.5%)	2.7	(0.1%, 5.3%)	0.5	(0%, 1.8%)	0	(0%, 0.3%)	–1.6	1.38
12	(144–155)	189	18	(12.2%, 23.7%)	40.2	(33%, 47.5%)	3.2	(0.4%, 5.9%)	1.1	(0%, 2.8%)	0.5	(0%, 1.8%)	–1.72	1.39
Total (6–12)	(72–155)	1319	18.7	(16.5%, 20.8%)	39.3	(36.6%, 41.9%)	3.3	(2.3%, 4.3%)	1.1	(0.5%, 1.7%)	0.2	(0%, 0.5%)	−1.66	1.45
**Both Sexes combined**														
6	(72–83)	356	21.9	(17.5%, 26.3%)	43.5	(38.2%, 48.8%)	6.5	(3.8%, 9.2%)	4.5	(2.2%, 6.8%)	2.2	(0.6%, 3.9%)	–1.64	1.73
7	(84–95)	363	22.6	(18.1%, 27%)	44.1	(38.8%, 49.3%)	2.8	(0.9%, 4.6%)	0.8	(0%, 1.9%)	0.6	(0%, 1.5%)	–1.84	1.51
8	(96–107)	379	24	(19.6%, 28.4%)	45.1	(40%, 50.3%)	3.4	(1.5%, 5.4%)	1.6	(0.2%, 3%)	0.3	(0%, 0.9%)	–1.86	1.55
9	(108–119)	372	20.2	(15.9%, 24.4%)	40.9	(35.7%, 46%)	5.6	(3.2%, 8.1%)	2.2	(0.5%, 3.8%)	1.3	(0%, 2.6%)	–1.65	1.67
10	(120–131)	378	19	(15%, 23.1%)	39.9	(34.9%, 45%)	4.5	(2.3%, 6.7%)	1.9	(0.4%, 3.3%)	0.5	(0%, 1.4%)	–1.6	1.58
11	(132–143)	375	19.5	(15.3%, 23.6%)	41.3	(36.2%, 46.5%)	3.7	(1.7%, 5.8%)	1.1	(0%, 2.2%)	0.5	(0%, 1.4%)	–1.7	1.47
12	(144–155)	379	22.4	(18.1%, 26.8%)	46.2	(41%, 51.3%)	3.4	(1.5%, 5.4%)	0.8	(0%, 1.8%)	0.3	(0%, 0.9%)	–1.84	1.45
Total (6–12)	(72–155)	2602	21.4	(19.8%, 23%)	43	(41.1%, 44.9%)	4.3	(3.5%, 5.1%)	1.8	(1.3%, 2.3%)	0.8	(0.4%, 1.2%)	−1.73	1.57

1 Includes children who are below −3SD from the WHO Child Growth Standards.

### Geographical/cluster distribution

The geographical distribution of WAZ score <-2SD was high in Lingasugur taluka at 48.9% (95% CI: 43.8%−54.0%), followed by Devadurga at 46.5% (95% CI: 40.5%−52.6%), Manvi at 45.1% (95% CI: 40.0%−50.3%), Raichur at 43.5% (95% CI: 37.9%−49.1%), and Sindhanur at 40.9% (95% CI: 34.8–46.9). The distribution of HAZ score <-2SD was more in Devadurga taluka at 34.6% (95% CI: 30.1%−39.1%), and it was the least in Manvi at 20.0% (95% CI: 16.8%−23.2%). Among all the districts, the prevalence of thinness BMI-Z scores <-2SD was high in Manvi taluka at 49.4% (95% CI: 45.3%−53.4%) followed by Lingasugur taluka at 46.5% (95% CI: 42.4%−50.6%) and other talukas of Raichur district. Severe thinness (< −3SD) was found highly prevalent in Lingasugur, 27.5% (95% CI: 23.8%−31.2%), compared with Manvi taluka, 22.9% (95% CI: 19.5%−26.3%). One of the interesting findings in Sindhanur taluka was the very high prevalence of overweight [(7.2%) (95% CI: 4.6%−9.8%)] and obesity [(3.7%) (1.8%−5.6%)] compared with the rest of the talukas in the district (Table 5).

**Table 5:** Cluster-wise distribution of weight-for-age, height-for-age, and BMI-for-age among the school children of age 6–12 years in the rural areas of Raichur district, Karnataka, India.

**Cluster**		**Weight–for–age** ^**^ **(%)**											
	**N**	**%** < −**3SD**	**(95% CI)**	**%** < −**2SD**^1^	**(95% CI)**	**Mean**	**SD**						
Manvi	359	15.6	(11.9,19.4)	45.1	(40.0, 50.3)	−1.76	1.18						
Devadurga	260	19.2	(14.4, 24.0)	46.5	(40.5, 52.6)	−1.9	1.21						
Lingasur	368	21.5	(17.3, 25.7)	48.9	(43.8, 54.0)	−1.94	1.37						
Raichur	301	15.9	(11.8, 20.1)	43.5	(37.9, 49.1)	−1.82	1.17						
Sindhanur	252	16.3	(11.7, 20.1)	40.9	(34.8, 46.9)	−1.7	1.36						
Total	1540	17.8	(15.8%, 19.7%)	45.3	(42.7%, 47.8%)	−1.83	1.27						
**Height–for–age (%)**													
		**%** < −**3SD**	**(95% CI)**	**%** < −**2SD**^1^	**(95% CI)**	**Mean**	**SD**						
Manvi	630	5.2	(3.4%, 7.1%)	20	(16.8%, 23.2%)	−0.99	1.3						
Devadurga	448	10.3	(7.3%, 13.2%)	34.6	(30.1%, 39.1%)	−1.43	1.42						
Lingasur	629	5.6	(3.7%, 7.4%)	26.4	(22.9%, 29.9%)	−1.13	1.34						
Raichur	540	5.4	(3.4%, 7.4%)	28	(24.1%, 31.8%)	−1.31	1.16						
Sindhanur	441	14.1	(10.7%, 17.4%)	29.9	(25.5%, 34.3%)	−1.45	1.47						
Total	2688	7.6	(6.6%, 8.6%)	27.2	(25.5%, 28.9%)	−1.24	1.34						
**BMI–for–age (%)**													
		**%** < −**3SD**	**(95% CI)**	**%** < −**2SD**^1^	**(95% CI)**	**%** > +**1SD**	**(95% CI)**	**%** > +**2SD**	**(95% CI)**	**%** > +**3SD**	**(95% CI)**	**Mean**	**SD**
Manvi	620	22.9	(19.5%, 26.3%)	49.4	(45.3%, 53.4%)	1.8	(0.7%, 2.9%)	0.3	(0%, 0.8%)	0	(0%, 0.1%)	−1.95	1.36
Devadurga	431	15.8	(12.2%, 19.3%)	39.2	(34.5%, 43.9%)	4.4	(2.4%, 6.5%)	0.7	(0%, 1.6%)	0	(0%, 0.1%)	−1.61	1.45
Lingasur	593	27.5	(23.8%, 31.2%)	46.5	(42.4%, 50.6%)	3.5	(2%, 5.1%)	1.2	(0.2%, 2.1%)	0.7	(0%, 1.4%)	−1.91	1.62
Raichur	528	20.1	(16.6%, 23.6%)	41.3	(37%, 45.6%)	3	(1.5%, 4.6%)	0.8	(0%, 1.6%)	0.2	(0%, 0.7%)	−1.71	1.47
Sindhanur	430	17.9	(14.2%, 21.6%)	34.9	(30.3%, 39.5%)	10.2	(7.3%, 13.2%)	7.2	(4.6%, 9.8%)	3.7	(1.8%, 5.6%)	−1.32	1.89
Total	2602	21.4	(19.8%, 23%)	43	(41.1%, 44.9%)	4.3	(3.5%, 5.1%)	1.8	(1.3%, 2.3%)	0.8	(0.4%, 1.2%)	−1.73	1.57

1 Includes children who are below −3SD from the WHO Child Growth Standards,

** Weight-for-age reference data are not available beyond 10 years because this indicator does not distinguish between height and body mass in an age period where many children are experiencing the pubertal growth spurt and may appear as having excess weight (by weight-for-age) when in fact they are just tall.

## Discussion

The NFHS-5 (2019–2020) findings from the Raichur district have highlighted a high prevalence of stunting (39.8%), wasting (23.2%), and underweight (40.7%) in the under-5 age group (28). The lack of nutritional data in school-age children from one of the most backward districts of Karnataka stressed the immediate need to bridge the gap. Thus, the current study aimed at generating evidence to devise holistic interventions in future. The results of the present study illustrated the perpetuation of undernutrition in school-going children.

The present study found an overall high prevalence of underweight (45.3%), with 17.8% of the children being severely underweight, with no significant difference between the genders. The findings of this study suggest that the prevalence of underweight in the study area was higher compared with the overall prevalence of Karnataka reported in the Comprehensive National Nutritional Survey (CNNS) 2019. According to CNNS data for the age group of 5–9 years, the distribution of WAZ less than−2SD was 35.2% and 39.5% in India and Karnataka, respectively, whereas this study showed that in the Raichur district, it was 45.3%. Similarly, the percentage below−3SD was 10.0% at the national level, 11.3% at the state level (27), and 17.8% at the study district. One of the studies conducted in the Mandya district of Karnataka reported the underweight to be 30.3%, which was significantly less compared with this study's results (30). A study conducted in Telangana, a neighboring state of Karnataka, had reported 30.85% underweight (31), and a study conducted in Punjab found that 58.8% of children (5–9 years) were severely underweight (10). This study showed a higher prevalence of underweight than studies conducted in Sri Lanka, Nigeria, and Nepal (7, 32, 33) and a lower prevalence than a study conducted in Ethiopia, where the prevalence was 59.7% (34). The underweight results of this study mirror the results of the study in Ghana, where the prevalence was 45.8% (35). The studies conducted by Isanaka et al. (36) in Colombia and Ieiri et al. (37) in the Philippines have established a relationship between the underweight and food insecurity, utilizing underweight as a nutritional indicator to assess recent weight loss or taking into consideration people who are unable to gain weight because of a lack of food supply or intake due to episodes of disease (38, 39). The findings of this study make way to explore the challenges associated with food insecurity in the Raichur district, where most of the children mainly depend on mid-day meals to cover most of their daily calorie requirements. The Mid-Day Meal Scheme is a government-sponsored program in India that aims to improve the nutritional status among primary and upper primary school children by providing free mid-day meals, meeting the minimum nutritional standard of 450–700 kCal and 12–20 g of protein (40).

Another notable finding of this study was the left-side skewness of the HAZ kernel density plots compared with the WHO growth standard graph, indicating the high prevalence of stunting. The distribution of stunting in the study area was more when compared with that of Karnataka (27). The study results align with the findings of the studies conducted in the south Indian districts of Mandya and Karimnagar (30, 31); however, higher compared with the study in Sri Lanka (32) and Nigeria (7) and much lower compared with the study undertaken in Ethiopia by Herrador et al. (41). According to the report of NFHS-5 data and the Raichur district nutrition profile carried out in 2020 by the International Food Policy Research Institute (IFPRI), the prevalence of stunting was approximately 40% among < 5-year-old children (28, 42), indicating that the interventions to address undernutrition have not yielded the expected results in the aspiration district. In this study, the distribution of stunting was found more in female children compared with male children; a similar trend was observed in the study conducted by Nowsin et al. (43) in Bangladesh; in contrast, the study in Srilanka by Naotunna et al. (32) found a higher distribution of stunting in male children than female children. Another study from Abakaliki metropolis of Nigeria found no difference in the stunting proportion between the genders (7). Stunting was most prevalent in the age group of 144–155 months, with a significantly high occurrence among female children compared with male children. The study conducted by Bongale Y T et al. observed that children aged 10–12 years had a 70% higher likelihood of experiencing stunting compared with children aged 6–7 years. One of the reasons could be prolonged exposure to chronic food shortages (44). The finding of higher prevalence among female children could be linked to the factors such as household dynamics, gender bias, and parental preferences, favoring male children (45). Several studies have found that mothers' educational status, poor dietary diversity, household income, and size were significantly associated with stunting (5, 46, 47). Since Raichur is one of the most socioeconomically underdeveloped districts, it is important to identify the causes of stunting, especially in female children because the effects of stunting tend to be passed down from generation to generation and have an impact on pregnancy outcomes such as small for gestational age and preterm birth (48).

The high prevalence of thinness indicates acute undernutrition, perhaps due to an acute shortage in the supply or intake of food (7). This study found a significantly higher prevalence of thinness (43%) than the overall prevalence in India (23.0%) and Karnataka (28.2%) (27). The distribution of thinness was more in male children when compared with female children. One reason could be gender disparities in physical activity levels, contributing to differences in energy expenditure between male and female children. According to a longitudinal study, male children demonstrated a daily increase in local energy expenditure from the age of 5 to 10 years. In contrast, female children experienced a significant 50% decline in physical activity between the age of 6 and 9 years (49, 50). There may be various sociocultural and psychosocial factors that influence the socialization of girls toward physical activity, necessitating further exploration. Similar findings were observed in the study conducted in Sudan, Nigeria, Sri Lanka, and Myanmar (7, 32, 51, 52). One of the interesting findings of the study was the geographical variation in the distribution of BMI for age Z-scores, particularly with the Sindhanur sub-district/taluka, where the prevalence of > +1SD and > +2SD was high compared with that of other talukas in the district as well as that of Karnataka state (27). Sindhanur as a major taluka with thriving commercial and industrial activities (53) may have better socioeconomic conditions, explaining the variation compared with other talukas. This finding implies that the interventions should be segmented and much more comprehensive to tackle both thinness and overweight in the target talukas facing the double burden of malnutrition.

Considering this study among some of the limited studies conducted in India regarding malnutrition in school-age children, the authors acknowledge some limitations in this study. The study results should be interpreted after careful contemplation of the methodology. As the study mainly focused on the children of rural areas, the study's findings are valid, particularly for rural settings. Even though adequately trained doctors from RIMS did the anthropometric measurements, some anthropometric measurement errors attributed to inter-observer and intra-observer bias cannot be denied in the study.

## Conclusion

In conclusion, the Raichur district, which is working to catch up with the rest of the country in terms of key development indicators, has a serious problem with undernutrition among school-going children (6–12 years). Additionally, exploratory studies are advised in this age group to break the intergenerational cycle of undernutrition and plan an evidence-based intervention by identifying the factors linked to undernutrition.

## Data availability statement

The raw data supporting the conclusions of this article will be made available by the authors, without undue reservation.

## Ethics statement

The studies involving human participants were reviewed and approved by Institutional Ethics Committee of Raichur Institute of Medical Sciences (RIMS), with approval number RIMS/IEC/Approval/02/22-23. Written informed consent to participate in this study was provided by the participants' legal guardian/next of kin.

## Author contributions

PV: project implementation, management, data collection, anthropometry, BMI analysis, and the first draft of the manuscript. SN: project implementation, management, data collection, and the first draft of the manuscript. UP: project implementation, management, data collection, anthropometry, data entry, and the first draft of the manuscript. RK: project implementation, fieldwork management, and finalization of the draft. NC and MB: data review, analysis, drafting, and finalizing the manuscript. SH and SS: project implementation, fieldwork management, and finalization of the draft. SR: conceptualization, overall project management, drafting, and finalization of the manuscript. All authors contributed to the article and approved the submitted version.

## References

[1] De Onis M Blossner M Borghi E. Prevalence and trends of stunting among preschool children, 1990–2020. Public Health Nutr. (2012) 15:142–8. 10.1017/S1368980011001315 21752311

[2] World Health Organization. Malnutrition. (2021). Available at: https://www.who.int/news-room/fact-sheets/detail/malnutrition (accessed January 14, 2023).

[3] National Family Health Survey India (NFHS-5). (2022). Available at: http://rchiips.org/nfhs/index.shtml (Accessed on 14 January 2023).

[4] Government of India. Press information Bureau, Cabinet Approves setting up of National Nutrition Mission. (2017). Available at: https://pib.gov.in/newsite/printrelease.aspx?relid=174025 (accessed January 16, 2023).

[5] Rayhan SK Banerjee A Rana MJ. Nutritional status and concomitant factors of stunting among preschool children in Malda, India: A micro-level study using a multilevel approach. BMC, Public Health. (2021) 21:1–13. 10.1186/s12889-021-11704-w 34530789 PMC8447797

[6] Srivastava A Mahmood SE Srivastava PM Shrotriya VP Kumar B. Nutritional status of school-age children - A scenario of urban slums in India. Arch Public Heal. (2012) 17:1–8. 10.1186/0778-7367-70-8 22958757 PMC3436633

[7] Umeokonkwo AA Ibekwe MU Umeokonkwo CD Okike CO Ezeanosike OB Ibe BC. Nutritional status of school-age children in Abakaliki metropolis, Ebonyi State, Nigeria. BMC Pediatr. (2020) 20:1–9. 10.1186/s12887-020-1994-5 32145745 PMC7060553

[8] Lundeen EA Behrman JR Crookston BT Dearden KA Engle P Georgiadis A. Growth faltering and recovery in children aged 1-8 years in four low- and middle-income countries: Young Lives. Public Health Nutr. (2014) 17:2131–7. 10.1017/S1368980013003017 24477079 PMC4043952

[9] Kuther T. Physical and Cognitive Development in Early Childhood. Lifespan Development: Lives in Context. Western Connecticut: SAGE Publications, Inc (2019). 816 p.

[10] Verma M Sharma P Khanna P Srivastava R Sahoo SS. Nutrition status of school children in Punjab, India: findings from school health surveys. J Trop Pediatr. (2021) 67: 1-11 fmaa068. 10.1093/tropej/fmaa068 33130876

[11] Black RE Victora C Walker SP Bhutta ZA Christian P de Onis M. Maternal and child undernutrition and overweight in low-income and middle-income countries. Lancet. (2013) 382:427–51. 10.1016/S0140-6736(13)60937-X 23746772

[12] United Nations. Millennium Development Goals. (2015). Available online at: https://www.un.org/millenniumgoals/ (accessed January 16, 2023).

[13] United Nations. Sustainable Development Goals. (2022). Available online at: https://www.un.org/sustainabledevelopment/health/ (accessed January 16, 2023).

[14] Das JK Lassi ZS Hoodbhoy Z Salam RA. Nutrition for the next generation: older children and adolescents. Ann Nutr Metab. (2018) 72:56–64. 10.1159/000487385 29631269

[15] Coly AN Milet J Diallo A Ndiaye T Bénéfice E Simondon F. Preschool stunting, adolescent migration, catch-up growth, and adult height in young Senegalese men and women of rural origin. J Nutr. (2006) 136:2412–20. 10.1093/jn/136.9.2412 16920863

[16] Tebeje DB Agitew G Mengistu NW Aychiluhm SB. Under-nutrition and its determinants among school-aged children in northwest Ethiopia. Heliyon. (2022) 8:1–9. 10.1016/j.heliyon.2022.e11235 36339772 PMC9626546

[17] Best C Neufingerl N Van Geel L Van Den Briel TVD Osendarp S. The nutritional status of school-aged children: why should we care? Food Nutr Bull. (2010) 31:400–17. 10.1177/156482651003100303 20973461

[18] Prentice AM Ward KA Goldberg GR Jarjou LM Moore SE Fulford AJ. Critical windows for nutritional interventions against stunting. Am J Clin Nutr. (2013) 97:911–8. 10.3945/ajcn.112.052332 23553163 PMC3628381

[19] Hemalatha R Radhakrishna K Kumar BN. Undernutrition in children & critical windows of opportunity in Indian context. Indian J Med Res. (2018) 148:612–20. 10.4103/ijmr.IJMR_1963_18 30666986 PMC6366257

[20] De Onis M Onyango AW Borghi E Borghi E Siyam A Nishida C. Siekmann, J. Development of a WHO growth reference for school-aged children and adolescents. Bull World Health Organ. (2007) 85:660–7. 10.2471/BLT.07.043497 18026621 PMC2636412

[21] Rathi K Kamboj P Gupta Bansal P Toteja GN. A review of selected nutrition and health surveys in India. Indian J Med Res. (2018) 148:596–611. 10.4103/ijmr.IJMR_1808_18 30666985 PMC6366271

[22] Kinyoki DK Ross JM Atwood AL Munro SB Schaeffer LE Mahdieh AF et al. Mapping local patterns of childhood overweight and wasting in lowand middle-income countries between 2000 and 2017. Nat Med. (2020) 26: 750–9. 10.1038/s41591-020-0807-6 32313249 PMC7220891

[23] NITI Aayog. DEEP DIVE, Insights from Champions of Change, The Aspirational Districts Dashboard. (2018). Available online at: https://www.niti.gov.in/sites/default/files/2018-12/FirstDeltaRanking-May2018-AspirationalRanking.pdf (accessed January 16, 2023).

[24] NITI Aayog. Aspirational Districts Programme. (2018). Available online at: https://www.niti.gov.in/aspirational-districts-programme (accessed January 16, 2023).

[25] Raichur District. District at a Glance. (2023). Available online at: https://raichur.nic.in/en/ (accessed May 31, 2023).

[26] NITI Aayog. India, National Multidimensional Poverty Index, Baseline Report. (2021). Available online at: https://www.niti.gov.in/sites/default/files/2021-11/National_MPI_India-11242021.pdf (accessed May 31, 2023).

[27] Ministry of Health Family Welfare. National Family Health Survey-5, District Fact Sheet, Raichur, Karnataka. (2022). Available online at: http://rchiips.org/nfhs/NFHS-5_FCTS/KA/Raichur.pdf (accessed June 11, 2023).

[28] Ministry of Health Family Welfare UNICEF, Population, Council. Comprehensive National Nutrition Survey. (2019). Available at: https://nhm.gov.in/WriteReadData/l892s/1405796031571201348.pdf (Accessed January 18, 2023).

[29] National Health Mission. Revised Policy Guidelines on National Iodine Deficiency Disorders Control Programme. (2006). Available online at: https://nhm.gov.in/images/pdf/programmes/ndcp/niddcp/revised_guidelines.pdf (accessed December 18, 2023).

[30] Shivaprakash NC. Joseph, RB. Nutritional status of rural school-going children (6-12 years) of Mandya District, Karnataka. Int J Sci Study. (2014) 2:39–43.

[31] Shaikh M Kamble N Bhawnani D Bele S Rao SR. Assessment of nutritional status among school children of Karimnagar, Telangana, India. Int J Res Med Sci. (2016) 4:4611–7. 10.18203/2320-6012.ijrms20163340

[32] Naotunna NPGCR Dayarathna M Maheshi H Amarasinghe GS Kithmini VS Rathnayaka M. Nutritional status among primary school children in rural Sri Lanka: a public health challenge for a country with high child health standards. BMC Public Health. (2017) 17:1–11. 10.1186/s12889-016-4001-1 28068960 PMC5223320

[33] Joshi HS Gupta R Joshi MC. Mahajan V. Determinants of Nutritional Status of School Children - A Cross-Sectional Study in the Western Region of Nepal. NJIRM. (2011) 2:10–5.

[34] Mekonnen H Tadesse T Kisi T. Malnutrition and its Correlates among Rural Primary School Children of Fogera District, Northwest Ethiopia. J Nutr Disorders Ther. (2013). S12:2. 10.4172/2161-0509.S12-002

[35] Danquah AO. Nutritional status of upper primary school pupils in a rural setting in Ghana. Int J Nutr Food Sci. (2013) 2:320–6. 10.11648/j.ijnfs.20130206.19

[36] Isanaka S Mora-Plazas M Lopez-Arana S Baylin A Villamor E. Food insecurity is highly prevalent and predicts underweight but not overweight in adults and school children from Bogotá, Colombia. J Nutr. (2007) 137:2747–55. 10.1093/jn/137.12.2747 18029494

[37] Ieiri MCA Kosaka S Tomitsuka E Umezaki M. Factors affecting undernutrition among school children in Cebu, Philippines. Ecol Food Nutr. (2021) 60:182–97. 10.1080/03670244.2020.1813733 33035433

[38] Engle PL Lhotska L Armstrong H. The Care Initiative: Assessment Analysis and Action to Improve Care of Nutrition. New York, NY: UNICEF (1997). pp. 1–12.

[39] Mwaniki EW Makokha AN Muttunga JN. Nutrition status and associated morbidity risk factors among orphanage and non-orphanage children in selected public primary schools within Dagoretti, Nairobi, Kenya. East Afr Med J. (2014) 91:289–97. 26866080

[40] Department of School Education and Government of Karnataka MID DAY, MEALS. (2023). Available online at: https://www.schooleducation.kar.nic.in/mms/food.html (accessed May 31, 2023).

[41] Herrador Z Sordo L Gadisa E Moreno J Nieto J. Benito A et al. Cross-sectional study of malnutrition and associated factors among school aged children in rural and urban settings of Fogera and Libo Kemkem districts Ethiopia. Plos ONE. (2014) 29:1–11. 10.1371/journal.pone.0105880 25265481 PMC4179248

[42] NITI Aayog. District Nutrition Profile, Raichur-Karnataka. (2022). Available online at: https://www.niti.gov.in/sites/default/files/2022-07/Raichur-Karnataka.pdf (accessed December 18, 2022).

[43] Nowsin I Begum N Akbar E. Bin Alam MM. Study on nutritional status of rural school children of Bangladesh Bangladesh. J Physiol Pharmacol. (2014) 30:6–10. 10.3329/bjpp.v30i1.20780

[44] Bogale TY Bala ET Tadesse M Asamoah BO. Prevalence and associated factors for stunting among 6–12 years old school age children from rural community of Humbo district, Southern Ethiopia. BMC Public Health. (2018) 18:1–18. 10.1186/s12889-018-5561-z 29793479 PMC5968559

[45] Senbanjo IO Oshikoya KA Odusanya OO Njokanma OF. Prevalence of and risk factors for stunting among school children and adolescents in Abeokuta, southwest Nigeria. J Health Popul Nutr. (2011) 29:364–70. 10.3329/jhpn.v29i4.8452 21957675 PMC3190367

[46] Assemie MA Alamneh AA Ketema DB Adem AM Desita M Petrucka P et al. High burden of undernutrition among primary school-aged children and its determinant factors in Ethiopia; a systematic review and meta-analysis. Ital J Pediatr. (2020) 46:1–14. 10.1186/s13052-020-00881-w 32847566 PMC7448995

[47] Corsi DJ Mejía-Guevara I Subramanian SV. Risk factors for chronic undernutrition among children in India: Estimating relative importance, population attributable risk and fractions. Soc Sci Med. (2016) 157:165–85. 10.1016/j.socscimed.2015.11.014 26625852

[48] Kozuki N Katz J Lee AC Joshua PV Mariangela FS Ayesha S et al. Child Health Epidemiology Reference Group Small-for-Gestational Age/Preterm Birth Working Group. Short maternal stature increases risk of small-for-gestational-age and preterm births in low- and middle-income countries: individual participant data meta-analysis and population attributable fraction. J Nutr. (2015) 145:2542–50. 10.3945/jn.115.216374 26423738 PMC6457093

[49] Sweeting HN. Gendered dimensions of obesity in childhood and adolescence. Nutr J. (2008) 7:1–14. 10.1186/1475-2891-7-1 18194542 PMC2265740

[50] Molnár D Livingstone B. Physical activity in relation to overweight and obesity in children and adolescents. Eur J Pediatr. (2000) 159:S45–55. 10.1007/PL00014365 11011955

[51] Mohamed S Hussein MD. Prevalence of thinness, stunting and anemia among rural school-aged Sudanese children: a cross-sectional study. J Trop Pediatr. (2015) 61:260–5. 10.1093/tropej/fmv028 25896992

[52] Prenkert M Ehnfors M. Growth data of underprivileged children living in rural areas of Chin State, Burma/Myanmar, compared to the WHO reference growth standards: AN observational study. BMJ Open. (2016) 6:1–12. 10.1136/bmjopen-2015-009119 26787249 PMC4735213

[53] Directorate of Municipal Administration. Sindhanur City Municipal Council. (2023). Available online at: http://www.sindhanurcity.mrc.gov.in/en (accessed May 31, 2023).

